# Impacto de la estrategia nacional contra la obesidad y diabetes en la incidencia de diabetes en México, 2005-2023

**DOI:** 10.1590/0102-311XES180725

**Published:** 2026-07-27

**Authors:** Laura Fernandez-Contreras, Carlos Saucedo-Sadi, Víctor Manuel Molina-Morejón

**Affiliations:** 1 Facultad de Contaduría y Administración, Universidad Autónoma de Coahuila, Coahuila, México.

**Keywords:** Diabetes Mellitus, Estrategias de Salud Nacionales, Tasa de Incidencia, Diabetes Mellitus, National Health Strategies, Incidence Rate, Diabetes Mellitus, Estratégias Nacionais de Saúde, Taxa de Incidência

## Abstract

El objetivo fue evaluar los cambios en las tendencias temporales de la incidencia de diabetes mellitus tipo 2 en la población adulta de México antes y después de la introducción de la Estrategia Nacional para la Prevención y el Control del Sobrepeso, la Obesidad y la Diabetes (ENPCSOD), a través del empleo de análisis de series temporales interrumpidas. Estudio ecológico longitudinal de serie temporal interrumpida con modelo de mínimos cuadrados generalizados que incorpora estructura ARMA(0,1). Se utilizaron los casos anuales reportados en los Anuarios de Morbilidad de la Secretaría de Salud (2005-2023) y tasas ajustadas por edad (población ≥ 20 años). Se realizaron análisis estratificados por sexo y grupos de edad (20-44, 45-59, ≥ 60 años). Tras la ENPCSOD se observó una disminución inmediata de 55,20 casos por 100.000 adultos (IC95%: -86,24; -24,15; p = 0,004), sin evidencia de una tendencia descendente sostenida en el periodo post-estrategia (pendiente post-ENPCSOD, β = 5,52; p = 0,180). La pandemia de COVID-19 se asoció con una caída aguda en el nivel (β = -109,04; p < 0,001). Los efectos fueron heterogéneos: en mujeres hubo un cambio de nivel mayor (β = -94,77; p = 0,007) y en el grupo ≥ 60 años la pendiente post-ENPCSOD mostró un incremento significativo (β = 42,54; p = 0,047). Los hallazgos indican una asociación temporal entre la introducción de la ENPCSOD y cambios en la tendencia de la incidencia, los cuales deben interpretarse con cautela dada la naturaleza ecológica del diseño.

## Abstract (en)

The aim was to evaluate the changes in the temporal trends of the incidence of type 2 diabetes mellitus in the adult population of Mexico before and after the introduction of the National Strategy Against Obesity and Diabetes (ENPCSOD), using interrupted time series analyses. Longitudinal ecological study of interrupted time series with generalized least squares model incorporating ARMA(0.1) structure. The annual cases reported in the Morbidity Yearbooks of the Ministry of Health (2005-2023) and age-adjusted rates (population ≥ 20 years) were used. Analyses were performed stratified by sex and age groups (20-44, 45-59, ≥ 60 years). After the ENPCSOD, an immediate decrease of 55.20 cases per 100,000 adults was observed (95%CI: -86.24; -24.15; p = 0.004), with no evidence of a sustained downward trend in the post-strategy period (post-ENPCSOD slope, β = 5.52; p = 0.180). The COVID-19 pandemic was associated with a sharp drop in the level (β = -109.04; p < 0,001). The effects were heterogeneous: in women there was a major change in level (β = -94.77; p = 0.007) and in the ≥ 60-year-old group the post-ENPCSOD slope showed a significant increase (β = 42.54; p = 0.047). The findings indicate a temporal association between the introduction of ENPCSOD and changes in incidence trend, both of which should be interpreted with caution given the ecological nature of the design.

## Resumo

O objetivo foi avaliar as mudanças nas tendências temporais da incidência de diabetes mellitus tipo 2 na população adulta do México antes e depois da introdução da Estratégia Nacional para Prevenção e Controle do Sobrepeso, Obesidade e Diabetes (ENPCSOD), por meio da análise de séries temporais interrompidas. Estudo ecológico longitudinal de séries temporais interrompidas com modelo de mínimos quadrados generalizados incorporando estrutura ARMA(0,1). Foram utilizados os casos anuais relatados nos Anuários de Morbidade da Secretaria de Saúde (2005-2023) e as taxas ajustadas por idade (população ≥ 20 anos). Foram realizadas análises estratificadas por sexo e faixa etária (20-44, 45-59, ≥ 60 anos). Após a implementação da ENPCSOD, observou-se uma diminuição imediata de -55,20 casos por 100 mil adultos (IC95%: -86,24; -24,15; p = 0,004), sem evidência de uma tendência descendente sustentada no período pós-estratégia (inclinação pós-ENPCSOD, β = 5,52; p = 0,180). A pandemia de COVID-19 foi associada a uma queda acentuada no nível (β = -109,04; p < 0,001). Os efeitos foram heterogêneos: nas mulheres houve uma mudança de nível maior (β = -94,77; p = 0,007) e no grupo ≥ 60 anos a inclinação pós-ENPCSOD apresentou um aumento significativo (β = 42,54; p = 0,047). Os resultados indicam uma associação temporal entre a introdução da ENPCSOD e as mudanças na tendência de incidência, o que deve ser interpretado com cautela dada a natureza ecológica do estudo.

## Introducción

La diabetes mellitus tipo 2 es una enfermedad metabólica crónica y compleja, reconocida como un importante problema de salud pública con rasgos epidémicos ^1^. A escala mundial, la incidencia de la diabetes pasó de 200 millones de personas en 1990 a 830 millones en 2022, con un crecimiento más acelerado en los países de ingresos bajos y medianos ^2^. En la región de las Américas, aproximadamente 112 millones de adultos padecen esta afección, una estadística que se ha triplicado desde 1990 ^3^.

En México, de acuerdo con la *Encuesta Nacional de Salud y Nutrición* 2023, el 18,4% de los adultos vive con esta enfermedad (12,4% diagnosticados y 6% sin diagnóstico), mientras que entre los mayores de 60 años la prevalencia alcanza 27,6% ^4^ ^,^ ^5^. Además, la diabetes mellitus tipo 2 representa la segunda causa de muerte en el país (151.019 defunciones en 2020) y una de las principales causas de años de vida saludable perdidos, lo que refleja un fuerte impacto económico y social ^6^ ^,^ ^7^ ^,^ ^8^.

En este contexto, numerosos países han trazado estrategias nacionales para la prevención y control de enfermedades no transmisibles (ENT). En la región de América Latina, México, Chile y Brasil han sido pioneros en el diseño de políticas públicas integrales para la prevención de diabetes, las cuales incluyen impuestos a bebidas azucaradas, etiquetado frontal de alimentos y prohibición de publicidad de alimentos con alto contenido calórico en horarios infantiles ^9^ ^,^ ^10^ ^,^ ^11^ ^,^ ^12^.

En respuesta a esta crisis de salud pública, el Gobierno Federal lanzó la Estrategia Nacional para la Prevención y el Control del Sobrepeso, la Obesidad y la Diabetes (ENPCSOD) en octubre de 2013, que constituyó una iniciativa de política pública intersectorial que abarcaba el impuesto de las bebidas azucaradas, la implementación del etiquetado frontal de los alimentos procesados y la regulación de la venta de alimentos en las instituciones educativas. Se concibió como un marco intersectorial que articula acciones de prevención, regulación del entorno alimentario, fortalecimiento de la atención médica y coordinación institucional. Su implementación fue progresiva y heterogénea, dependiendo de capacidades administrativas, recursos y prioridades sectoriales ^13^.

Si bien la mayoría de los estudios sobre la ENPCSOD y políticas afines en México se ha centrado en resultados intermedios, como patrones de compra, consumo y reformulación de productos, o en medidas de prevalencia, existe una limitada evidencia empírica sobre su impacto en desenlaces de salud poblacionales. En particular, son escasos los estudios que evalúan cambios en la incidencia de diabetes mellitus tipo 2 mediante diseños cuasiexperimentales robustos ^14^ ^,^ ^15^.

Aunque la diabetes mellitus tipo 2 es tradicionalmente abordada desde el enfoque biomédico como una emergencia epidemiológica, desde la perspectiva de la Salud Colectiva se entiende como un fenómeno social determinado por desigualdades estructurales, gobernanza fragmentada y dinámicas políticas. En México, la ENPCSOD representa más que una intervención clínica: es una política pública que articula impuestos a bebidas azucaradas, etiquetado frontal y regulación institucional, en un entorno marcado por debilidades institucionales, fragmentación territorial y limitada participación social ^16^. Comprender su impacto exige analizarla no solo como una serie de indicadores epidemiológicos, sino como un proceso de gobernanza en salud que opera en el espacio público, disciplinando la oferta e induciendo transformaciones sociales.

La ENPCSOD establece un esquema de evaluación con un horizonte de seis años y avances anuales para medir la efectividad de las acciones y ajustar su diseño y operación, orientado a desacelerar la prevalencia de sobrepeso y obesidad y reducir la carga de enfermedades no transmisibles, incluida la diabetes mellitus ^17^.

Desde esta perspectiva, el análisis de series temporales interrumpidas (ITS, por su sigla em inglés) constituye un enfoque particularmente adecuado para este propósito, ya que permite estimar de forma objetiva cambios inmediatos y sostenidos en indicadores poblacionales, distinguiéndolos de las tendencias históricas preexistentes en contextos donde no es factible contar con grupos comparadores ^18^ ^,^ ^19^ ^,^ ^20^. De este modo, el ITS no solo aporta rigor epidemiológico, sino que ofrece una herramienta empírica para examinar el impacto temporal de políticas públicas enmarcadas en procesos sociales y políticos más amplios.

Al sujetar resultados epidemiológicos con implicaciones administrativas, este estudio favorece el fortalecimiento de la toma de decisiones fundamentadas en evidencia en América Latina y el Caribe. Los hallazgos son pertinentes no solo en México, sino que proporcionan lecciones significativas para el diseño, implementación y evaluación de estrategias regionales destinadas a mitigar la carga de las ENT en contextos de alta vulnerabilidad y recursos limitados.

La ENPCSOD constituye un marco nacional de políticas y lineamientos estratégicos cuya implementación fue progresiva, heterogénea y dependiente de múltiples actores institucionales. En este contexto, el presente estudio no evalúa la ejecución específica de cada pilar de la Estrategia, sino que analiza si la introducción de este marco regulatorio se asocia temporalmente con cambios en la tendencia poblacional de incidencia de diabetes mellitus tipo 2.

Partimos de la hipótesis de que la implementación de la ENPCSOD se asocia temporalmente con modificaciones en el comportamiento de la serie de incidencia de diabetes mellitus tipo 2, incluyendo posibles cambios inmediatos atribuibles a factores operativos, institucionales o conductuales de corto plazo, así como variaciones en la tendencia a mediano plazo, más que a efectos biológicos inmediatos sobre la aparición de nuevos casos.

Por lo tanto, el objetivo general de este estudio es evaluar los cambios en las tendencias temporales de la incidencia de diabetes mellitus tipo 2 en la población adulta de México antes y después de la introducción de la ENPCSOD, a través del empleo de ITS.

## Método

Se llevó a cabo un estudio ecológico longitudinal y cuasiexperimental de ITS, para evaluar el impacto poblacional de la ENPCSOD sobre las tasas de incidencia de diabetes mellitus tipo 2 ajustadas por edad y desagregadas por grupos de edad y sexo en la población adulta mexicana (≥ 20 años). El universo quedó conformado por 7.748.602 adultos diagnosticados con diabetes mellitus tipo 2 en el periodo 2005-2023 en México.

### Fuentes de datos

Se utilizaron datos de los Anuarios de Morbilidad de México (https://epidemiologia.salud.gob.mx/anuario/html/index.html; 2005-2023). Se obtuvieron los casos nuevos anuales de diabetes mellitus tipo 2 en la población ≥ 20 años. Se consideraron como casos incidentes de diabetes mellitus tipo 2 aquellos registros notificados con el código de la 10ª revisión de la Clasificación Internacional de Enferemedades (CIE-10) correspondiente a diabetes mellitus tipo 2 en los anuarios.

En México, la diabetes mellitus tipo 2 se reporta a través del Sistema Nacional de Vigilancia Epidemiológica (SINAVE) mediante el formato SUIVE (Sistema Único de Información para la Vigilancia Epidemiológica) ^21^, que integra notificaciones de unidades médicas de instituciones públicas y privadas que conforman el Sistema Nacional de Salud, de acuerdo con la NOM-017-SSA2-2012 ^22^. Aunque esta vigilancia es obligatoria y estandarizada, la cobertura efectiva depende de la detección clínica y del acceso a servicios de salud; encuestas nacionales indican que una proporción significativa de personas con diabetes mellitus tipo 2 no está diagnosticada ^5^, lo que sugiere subregistro en las estadísticas oficiales de incidencia. Estos aspectos deben tenerse en cuenta al interpretar tendencias temporales basadas en registros notificados.

Para el cálculo de las tasas específicas anuales por edad se empleó como denominador la población total correspondiente a cada año. Por otro lado, para el cálculo de las tasas ajustadas por edad se aplicó el método directo de estandarización, donde se empleó como población estándar la población censal quinquenal de México del *Censo de Población y Vivienda 2010* ^23^. La tasa ajustada por edad se calculó mediante la fórmula de ajuste directo:

$$R_{adj}=\sum_{i=1}^{k}w_{i}∙r_{i}$$

donde *r*_*i*_ = *C*_*i*_ */N*_*i*_ es la tasa específica por persona en el grupo de edad *i*, y *w*_*i*_ es el peso de la población estándar en el mismo grupo. Las tasas se presentan por 100.000 habitantes multiplicando *R*_*adj*_ por 10^5^.

Esto permitió construir series anuales con tasas ajustadas por edad para la población adulta total (≥ 20 años), seguido de modelos estratificados por sexo y grupos de edad (20-44, 45-59 y ≥ 60 años).

### Análisis estadístico

Se evaluó la posible colinealidad entre las variables del modelo, mediante el cálculo del factor de inflación de varianza (VIF, por su sigla en inglés). Todos los VIF fueron inferiores a 5, lo que indica baja colinealidad y permite interpretar los coeficientes de forma independiente.

Se especificó una estructura de correlación ARMA(0,1) para el término de error en el modelo de mínimos cuadrados generalizados (GLS, por su sigla em inglés) con el fin de capturar la dependencia temporal de primer orden observada en la serie. La elección de MA(1) se basó en la inspección de función de autocorrelación y función de autocorrelación parcial (ACF/PACF, por sus siglas em inglés) de los residuos y en la comparación de especificaciones alternativas (AR(1), ARMA(1,1), modelos GLS sin correlación, mediante criterios de información de Akaike (AIC) y Bayesiano (BIC), seleccionándose la estructura que ofreció mejor ajuste sin sobreajuste. Este modelo se implementó en RStudio (http://www.r-project.org) mediante la función *gls*() del paquete *nlme*:

$$Y_{t}=\beta_{0}+\beta_{1}∙{Tiempo}_{t}+\beta_{2}∙Intervención+\beta_{3}∙{TiempoPostIntrvención}_{t}+{\beta_{4}*COVID+\beta_{5}*{\left(TiempoPostCOVID\right)}_{t}+\varepsilon }_{t}$$

Donde: *Y*_*t*_: tasa de incidencia de diabetes mellitus tipo 2 en el año *t*; *β*_*0*_: intercepto (nivel inicial en 2005); *β*_*1*_: pendiente preintervención (2005-2013); *β*_*2*_: cambio inmediato tras la ENPCSOD (2013); *β*_*3*_: cambio en la pendiente post-ENPCSOD; *β*_*4*_: cambio inmediato durante la pandemia por COVID-19 (2020); *β*_*5*_: cambio en la pendiente durante la pandemia; *ϵ*_*t*_: término de error con estructura ARMA(0,1).

La intervención ENPCSOD se codificó como 0 para los años ≤ 2013 y 1 para los años ≥ 2014, definiendo 2013 como el punto de interrupción del análisis. Este punto corresponde al inicio de la implementación progresiva de medidas regulatorias y programáticas alineadas con la Estrategia, concebida con evaluación anual y un horizonte de seis años. La operacionalización incluyó políticas efectivamente implementadas, como los lineamientos de alimentos en escuelas y el impuesto a bebidas azucaradas (2014), así como procesos regulatorios posteriores de etiquetado y la NOM-051 (Material Suplementario; https://cadernos.ensp.fiocruz.br/static//arquivo/supl-e00180725_9533.pdf). Este enfoque permite evaluar asociaciones temporales en la incidencia de diabetes mellitus tipo 2 sin atribuir efectos causales a componentes específicos.

Se incorporó la pandemia como segunda interrupción (2020) debido a su efecto disruptivo en la atención y registro de enfermedades crónicas. Se utilizó RStudio versión 4.3.1 con los paquetes *nlme*, *lmtest*, *car*, *forecast*, *AICcmodavg*, *ggplot2*, *purrr* y *MuMIn*.

### Validación del modelo y análisis de sensibilidad

Se verificó la estacionariedad de la serie y la ausencia de autocorrelación residual mediante el test de Ljung-Box y gráficos de ACF. Para evaluar homocedasticidad se realizó la prueba Breusch-Pagan. Se establecieron a priori como criterios de aceptabilidad: p ≥ 0,05 en Ljung-Box, ausencia de picos significativos en ACF dentro de bandas 95%, p ≥ 0,05 en Breusch-Pagan, y VIF < 5 (alerta)/< 10 (umbral problemático). Todos los diagnósticos se presentan en el Material Suplementario (https://cadernos.ensp.fiocruz.br/static//arquivo/supl-e00180725_9533.pdf).

Se consideró aceptable el cumplimiento razonable de estos supuestos. Los coeficientes del modelo se reportaron con intervalos del 95% de confianza (IC95%).

Para estimar la magnitud de los cambios aplicables a las intervenciones, se calcularon medidas de impacto absoluto y relativo en años clave (2013, inicio de la ENPCSOD; 2020, inicio de la pandemia de COVID-19). El impacto absoluto se definió como la diferencia entre la incidencia predicha por el modelo y la incidencia contrafactual proyectada bajo el escenario de no intervención. El impacto relativo se obtuvo al expresar esa diferencia como porcentaje del valor contrafactual (Material Suplementario; https://cadernos.ensp.fiocruz.br/static//arquivo/supl-e00180725_9533.pdf). Estas medidas permiten cuantificar el impacto poblacional de la política en términos de casos evitados o adicionales, facilitando la interpretación práctica de los hallazgos para la evaluación de beneficios y daños en salud pública.

Para evaluar sensibilidad, se repitió el análisis excluyendo 2020 y 2021; estos resultados se presentan como Material Suplementario (https://cadernos.ensp.fiocruz.br/static//arquivo/supl-e00180725_9533.pdf).

### Consideraciones éticas

El estudio utilizó datos secundarios, agregados, de dominio público y sin identificadores personales. Por ello, no requirió aprobación de un comité de ética ni consentimiento informado, de acuerdo con las pautas internacionales para investigaciones con datos secundarios.

## Resultados

En el período 2005-2023, las tasas ajustadas de incidencia de diabetes mellitus tipo 2 por edad en la población adulta mexicana, presentaron una tendencia descendente significativa antes de implementada la ENPCSOD, caracterizada por una reducción anual promedio de 7,15 casos por 100.000 adultos mayores de 20 años (IC95%: -11,863; -2,447; p = 0,011). Cabe señalar que la ENPCSOD se implementó en un contexto de tendencia previa no plenamente estable, por lo que los cambios estimados deben interpretarse como asociaciones temporales robustas en la serie, sin asumir una relación causal simple.

Tras la implementación de la estrategia en 2013, se observó un cambio inmediato en el nivel de la serie, marcado por una disminución estadísticamente significativa de 55,20 casos por 100.000 personas adultas (IC95%: -86,24; -24,15; p = 0,004), situándose por debajo de la proyección contrafactual. Sin embargo, el cambio en la pendiente posterior a la ENPCSOD no alcanzó significación estadística (β = 5,52; IC95%: -2,11; 13,14; p = 0,180).

**Figura 1:**
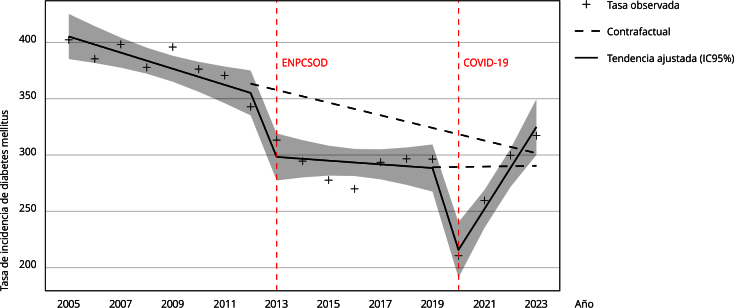
Tasa de incidencia (por 100.000 habitantes) ajustada por edad y predicción contrafactual de diabetes mellitus tipo 2. México, 2005-2023.

En 2020, el inicio de la pandemia COVID-19 se asoció con una disminución abrupta del nivel de la serie, de -109,04 casos por 100.000 adultos (IC95%: -147,97; -70,10; p < 0,001), seguida de un aumento significativo en la pendiente durante la pandemia (β = 38,00: IC95%: 23,62; 52,39; p < 0,001), sobrepasando el contrafactual (Figura 1 y Tabla 1).

ENPCSOD: Estrategia Nacional para la Prevención y el Control del Sobrepeso, la Obesidad y la Diabetes; IC95%: intervalo del 95% de confianza.

**Tabla 1:** Análisis de series de tiempo interrumpidas para la diabetes mellitus tipo 2. México, 2005-2023.

Coeficiente	Estimación	Error estándar	t	Valor de p	IC95%
Intercepto	412,36	12,27	33,60	< 0,001	388,30; 436,42
Tiempo	-7,15	2,40	-2,98	0,011	-11,86; -2,45
Intervención ENPCSOD	-55,20	15,84	-3,49	0,004	-86,24; -24,15
Pendiente post-ENPCSOD	5,52	3,89	1,42	0,180	-2,11; 13,14
Intervención COVID-19	-109,04	19,87	-5,49	< 0,001	-147,97; -70,10
Pendiente post-COVID-19	38,00	7,34	5,18	< 0,001	23,62; 52,39

ENPCSOD: Estrategia Nacional para la Prevención y el Control del Sobrepeso, la Obesidad y la Diabetes; IC95%: intervalo del 95% de confianza.

Fuente: elaboración propia.

**Figura 2:**
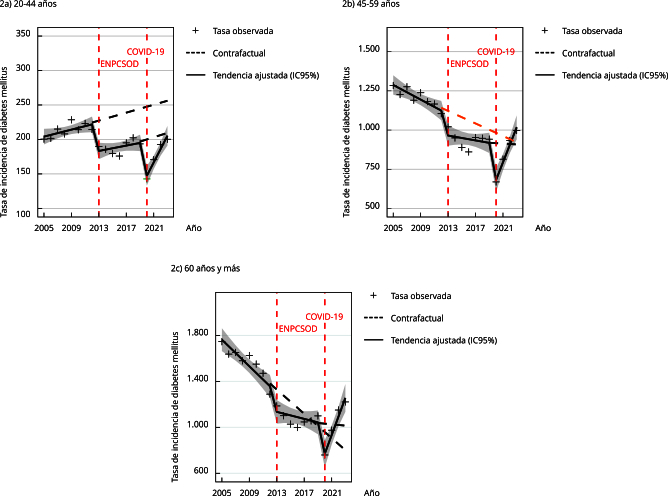
Tasa de incidencia (por 100.000 habitantes) específica por edad y predicción contrafactual de diabetes mellitus tipo 2. México, 2005-2023.

La Figura 2 y Tabla 2 presentan el análisis de ITS sobre las tasas de incidencia de diabetes mellitus tipo 2 específicas por grupos de edad. Se apreció que la pendiente antes de la ENPCSOD fue descendente y estadísticamente significativa en los grupos de edad de 45-59 años (β = -23,36; IC95%: -37,92; -8,79; p = 0,008) y 60 años y más (β = -57,83; IC95%: -81,06; -34,61; p < 0,001). Tras la implementación de la ENPCSOD se observó una reducción inmediata en las tasas ajustadas de incidencia de diabetes mellitus tipo 2. El cambio ascendente en la pendiente post-ENPCSOD fue significativo solo en el grupo de 60 años y más (β = 42,54; IC95%: 4,47; 80,60; p < 0,047).

La llegada de la COVID-19 representó un cambio abrupto y significativo en el nivel de la pendiente en los tres grupos etarios, con efecto más pronunciado en las personas mayores de 60 años (β = -434,73; IC95%: -616,75; -252,71; p < 0,001).

ENPCSOD: Estrategia Nacional para la Prevención y el Control del Sobrepeso, la Obesidad y la Diabetes; IC95%: intervalo del 95% de confianza.

**Tabla 2:** Análisis de series de tiempo interrumpidas para la diabetes mellitus tipo 2 por grupos de edad y sexo. México, 2005-2023.

Coeficiente	Estimación	Error estándar	t	Valor de p	IC95%
Grupos de edad (años)					
20-44					
Intercepto	201,55	6,96	28,95	< 0,001	187,90; 215,19
Tiempo	2,54	1,37	1,85	0,087	-0,15; 5,22
Intervención ENPCSOD	-40,47	9,25	-4,38	0,001	-58,59; -22,35
Pendiente post-ENPCSOD	-0,59	2,19	-0,27	0,792	-4,89; 3,71
Intervención COVID-19	-67,05	11,86	-5,66	< 0,001	-90,29; -43,81
Pendiente post-COVID-19	17,43	4,24	4,11	0,001	9,12; 25,73
45-59					
Intercepto	1311,17	37,93	34,57	< 0,001	1.236,82; 1.385,51
Tiempo	-23,36	7,43	-3,14	0,008	-37,92; -8,79
Intervención ENPCSOD	-151,63	49,15	-3,09	0,009	-247,96; -55,30
Pendiente post-ENPCSOD	15,56	12,01	1,30	0,218	-7,98; 39,10
Intervención COVID-19	-347,27	61,82	-5,62	< 0,001	-468,44; -226,10
Pendiente post-COVID-19	119,41	22,73	5,25	< 0,001	74,85; 163,97
≥ 60					
Intercepto	1820,79	61,01	29,85	< 0,001	1.701,22; 1.940,37
Tiempo	-57,83	11,85	-4,88	< 0,001	-81,06; -34,61
Intervención ENPCSOD	-208,04	75,74	-2,75	0,017	-356,48; -59,59
Pendiente post-ENPCSOD	42,54	19,42	2,19	0,047	4,47; 80,61
Intervención COVID-19	-434,73	92,87	-4,68	< 0,001	-616,75; -252,71
Pendiente post-COVID-19	177,31	35,87	4,94	< 0,001	107,01; 247,62
Sexo					
Femenino					
Intercepto	765,01	23,08	33,14	< 0,001	719,77; 810,26
Tiempo	-12,77	4,51	-2,83	0,014	-21,61; -3,94
Intervención ENPCSOD	-94,77	29,46	-3,22	0,007	-152,50; -37,03
Pendiente post-ENPCSOD	13,38	7,33	1,83	0,091	-0,98; 27,74
Intervención COVID-19	-232,62	36,69	-6,34	< 0,001	-304,53; -160,72
Pendiente post-COVID-19	84,72	13,73	6,17	< 0,001	57,81; 11,63
Masculino					
Intercepto	493,85	22,99	21,48	< 0,001	448,79; 538,91
Tiempo	4,65	4,50	1,03	0,320	-4,16; 13,46
Intervención ENPCSOD	-91,38	29,58	-3,09	0,009	-149,35; -33,40
Pendiente post-ENPCSOD	-2,52	7,29	-0,35	0,735	-16,80; 11,70
Intervención COVID-19	-163,35	37,03	-4,41	0,001	-235,94; -90,77
Pendiente post-COVID-19	52,71	13,73	3,84	0,002	25,80; 79,62

ENPCSOD: Estrategia Nacional para la Prevención y el Control del Sobrepeso, la Obesidad y la Diabetes; IC95%: intervalo del 95% de confianza.

Fuente: elaboración propia.

**Figura 3:**
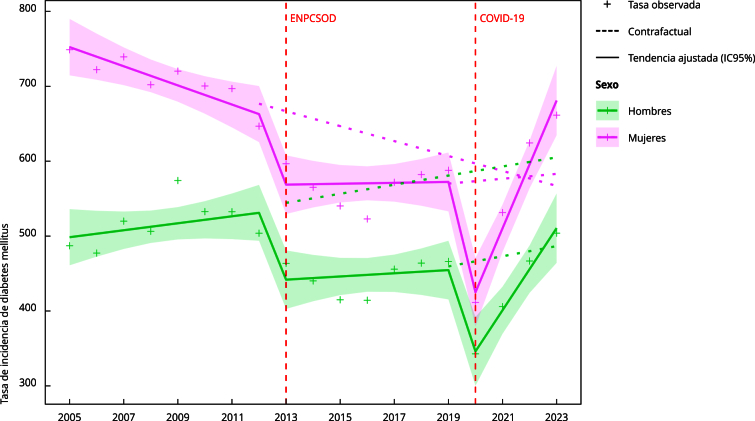
Tasa de incidencia (por 100.000 habitantes) específica por sexo y predicción contrafactual de diabetes mellitus tipo 2. México, 2005-2023.

Los resultados del estudio referente al análisis de ITS por sexo, reflejados en la Figura 3 y Tabla 2, evidenciaron que las tasas de incidencia de diabetes mellitus tipo 2 específicas para el sexo femenino tuvieron un comportamiento descendente significativo antes de la ENPCSOD (β = -12,77; IC95%: -21,61; -3,94; p = 0,014). La implementación de la estrategia se asoció con un cambio de nivel significativo (β = -94,77; IC95%: -152,50; -37,03; p = 0,007), seguida de un ascenso no significativo de la pendiente post-ENPCSOD (β = 13,38; IC95%: -0,98; 27,74; p = 0,091). La pandemia precipitó una disminución significativa del nivel (β = -232,62; IC95%: -304,53; -160,72), seguida de un aumento persistente de la pendiente a lo largo del período 2020-2023 (β = 84,72; IC95%: 57,81; 111,63; p < 0,001).

ENPCSOD: Estrategia Nacional para la Prevención y el Control del Sobrepeso, la Obesidad y la Diabetes; IC95%: intervalo del 95% de confianza.

Por otro lado, las tasas de incidencia específicas para el sexo masculino mostraron un comportamiento ascendente antes de trazada la ENPCSOD. La intervención se asoció con una reducción inmediata significativa de las tasas de incidencia de diabetes mellitus tipo 2 específicas para este sexo (β = -91,38; IC95%: -149,35; -33,40; p = 0,009), sin cambios significativos en la pendiente posterior. La llegada de la COVID-19 produjo un descenso abrupto estadísticamente significativo en el nivel de la pendiente (β = -163,35; IC95%: -235,94; -90,7; p = 0,001), seguido de un ascenso significativo de la pendiente durante el período pandémico (β = 52,71; IC95%: 25,80; 79,62; p = 0,002) (Figura 3 y Tabla 2).

## Discusión

La presente evaluación con ITS sobre la serie 2005-2023 aporta evidencia empírica nacional acerca de la asociación temporal entre la ENPCSOD y la dinámica de la incidencia de diabetes mellitus tipo 2 en México. La estrategia fue diseñada como un plan integral con acciones concurrentes en tres ejes: salud pública, atención médica y regulación sanitaria ^13^. El hallazgo de un cambio inmediato asociado a la introducción de la ENPCSOD sugiere que la introducción de la estrategia y las medidas reguladoras asociadas pudieron inducir cambios poblacionales detectables en un corto plazo.

Esta interpretación debe matizarse: la tendencia preintervención (β_1_ = -7,15) era ya descendente, por lo que parte del cambio de nivel observado puede corresponder a una intensificación de una dinámica preexistente y no exclusivamente a un efecto nuevo atribuible a la acción iniciada en 2013. Además, como se ha documentado en evaluaciones previas, medidas regulatorias implementadas en México, en particular el impuesto a bebidas azucaradas vigente desde enero de 2014, se asociaron rápidamente con cambios en patrones de compra y consumo, mecanismos plausibles para producir efectos tempranos en indicadores poblacionales ^24^.

Asimismo, dado que los datos analizados corresponden a casos diagnosticados y notificados, los resultados pueden reflejar variaciones en el acceso a los servicios de salud, en los procesos de registro o en la organización del sistema sanitario, y no exclusivamente cambios en el riesgo poblacional de desarrollar la enfermedad.

Debe considerarse que la reducción inmediata observada tras la introducción de la ENPCSOD no se tradujo en un cambio sostenido en la pendiente de la incidencia de diabetes mellitus tipo 2, lo que sugiere que los efectos iniciales no se consolidaron en el tiempo. Este patrón es consistente con evaluaciones previas de políticas nutricionales que muestran impactos tempranos en conductas y oferta alimentaria, pero una atenuación de los efectos poblacionales cuando la implementación es parcial, la intensidad regulatoria es limitada o no existe una coordinación intersectorial sostenida.

Asimismo, las tendencias observadas deben interpretarse considerando la coexistencia de otras políticas públicas y cambios estructurales durante el período de estudio, incluyendo modificaciones en la cobertura y organización de los servicios de salud, variaciones en la vigilancia y el registro de enfermedades crónicas, y la introducción o ajuste de medidas regulatorias no directamente vinculadas a la ENPCSOD. Estos factores pueden haber modificado tanto el riesgo real como la probabilidad de diagnóstico de diabetes mellitus tipo 2, contribuyendo a la pérdida de sostenibilidad del efecto inicial.

En el Caribe, políticas similares a la aplicada en Barbados confirmaron efectos inmediatos en ventas, aunque su sostenibilidad depende de marcos regulatorios armonizados, vigilancia adaptada y coordinación regional liderada por organismos como la Organización Panamericana de la Salud (OPS) y la Agencia de Salud Pública del Caribe (CARPHA, por su sigla en inglés) ^25^ ^,^ ^26^.

Los sistemas de etiquetado frontal y restricciones a publicidad han mostrado impactos rápidos en compras y composición de la oferta alimentaria. Las evaluaciones de políticas integradas en Chile, por ejemplo, documentaron reducciones en las compras de productos “high-in” superiores a las obtenidas por medidas aisladas. Estudios de seguimiento han reportado evidencias de reformulación y cambios persistentes en patrones de compra. Estas transformaciones en la disponibilidad y elección de productos alimentarios constituyen vías por las cuales las intervenciones pueden producir un cambio inmediato en indicadores poblacionales ^27^ ^,^ ^28^.

En Brasil, las políticas fiscales y de etiquetado muestran efectos heterogéneos condicionados por la gobernanza y la escala subnacional. La reducción del impuesto federal a bebidas azucaradas en 2016 y 2018 limitó su impacto, y los modelos sugieren que serían necesarias tasas más altas para reducir obesidad y diabetes. Aunque el país avanzó en el rotulado frontal entre 2020 y 2022, su diseño genera debate sobre efectividad. La baja magnitud fiscal, la implementación regulatoria parcial y la heterogeneidad territorial explican por qué los cambios de consumo a corto plazo no se traducen en reducciones sostenidas de incidencia sin medidas más ambiciosas y coordinadas ^29^ ^,^ ^30^.

La evidencia indica que los cambios en compras y oferta no siempre generan reducciones inmediatas y sostenidas en la incidencia de diabetes. Aunque los modelos proyectan beneficios a mediano y largo plazo, los estudios observacionales suelen no detectar descensos en 3-5 años, lo que coincide con un patrón de efectos tempranos seguidos de falta de consolidación, influido por tiempo insuficiente, magnitud limitada del cambio o factores contextuales como desigualdades y variaciones en diagnóstico o cobertura sanitaria ^31^.

Investigaciones como las de Vargas Bustamante et al. ^32^ y Munguía et al. ^33^ han documentado cambios en consumo de ultraprocesados y en la oferta, aunque sin evaluar directamente la incidencia de ENT. El estudio de Bueno Hernández ^34^ identificó que, pese a esfuerzos preventivos, la incidencia de diabetes mellitus tipo 2 siguió aumentando, atribuible a deficiencias institucionales, sociales y económicas. Nuestro trabajo complementa esa evidencia al cuantificar cambios de nivel y tendencia, reforzando que los avances iniciales no se consolidaron por limitaciones estructurales.

Desde una perspectiva de Salud Colectiva, nuestros hallazgos refuerzan la idea de que las intervenciones aisladas, aunque técnicamente sólidas, son frágiles sin una gobernanza intersectorial robusta. La ENPCSOD careció de mecanismos permanentes de coordinación entre sectores clave como salud, agricultura y educación, tal como menciona Calvillo et al. ^14^. Todo ello, limita su capacidad de producir transformaciones estructurales y sostenibles en la incidencia de diabetes mellitus tipo 2. Experiencias regionales muestran que políticas acompañadas de participación social y redes epistemológicas tienden a lograr impactos más duraderos en las ENT ^16^.

La sostenibilidad de impactos en salud derivados de políticas como la ENPCSOD está condicionada por determinantes sociales y capacidades institucionales. Desigualdades socioeconómicas y territoriales, barreras en el acceso a servicios de salud y la heterogeneidad en la implementación subnacional pueden limitar la traducción de cambios en el entorno alimentario en reducciones sostenidas de incidencia. Asimismo, la gobernanza intersectorial débil y la capacidad limitada para monitorear y escalar intervenciones pueden explicar por qué efectos tempranos en compra/oferta no se consolidaron en disminuciones prolongadas de incidencia ^35^.

La pandemia de COVID-19 representa un caso ilustrativo de cómo los determinantes sociales y los shocks sanitarios modifican patrones de incidencia y registro. Estudios internacionales han documentado disminuciones en diagnósticos durante 2020 seguidas de repuntes posteriores, atribuibles tanto a rezagos en detección como a cambios reales en riesgos metabólicos asociados al confinamiento, la dieta y la reducción de la actividad física ^36^ ^,^ ^37^ ^,^ ^38^. Además, investigaciones recientes sugieren que la infección por SARS-CoV-2 incrementa el riesgo de desarrollar diabetes mellitus, lo que refuerza la necesidad de vigilancia activa en la etapa postpandemia ^39^.

El incremento en la pendiente de la incidencia de diabetes mellitus tipo 2, observado tras la pandemia de COVID-19, particularmente en el grupo ≥ 60 años, requiere una interpretación cuidadosa. Este patrón podría reflejar un retraso acumulado en la detección durante los periodos de mayor interrupción de los servicios de salud, así como a un aumento real del riesgo metabólico asociado a confinamientos (menor actividad física, aumento de peso) y, potencialmente, a efectos directos o indirectos de la infección por SARS-CoV-2 sobre la glucorregulación ^40^ ^,^ ^41^. La coexistencia de ambos mecanismos es compatible con la magnitud y la persistencia de la tendencia ascendente observada en este grupo etario y subraya la necesidad de vigilancia activa y estudios de seguimiento.

Investigaciones en Europa, concluyen que una mayor implementación de políticas nutricionales y de actividad física no se asocia necesariamente con una menor prevalencia de diabetes. Las políticas implementadas hasta 2014 en la Unión Europea tuvieron cierto impacto en la reducción de la carga de diabetes, pero no lo suficiente como para cambiar esta tendencia al alza ^42^. Este aporte es especialmente relevante para América Latina y el Caribe, donde persisten desafíos comunes en la evaluación de políticas públicas y en la sostenibilidad de intervenciones preventivas.

La heterogeneidad observada - con un mayor cambio inmediato de nivel en mujeres y comportamientos diferenciados por grupos etarios, así como un incremento de la pendiente en ≥ 60 años - sugiere efectos diferenciados según los determinantes sociales, el acceso a los servicios y la cobertura de los programas comunitarios. La mayor reducción en mujeres puede estar relacionada con una mayor cobertura en programas comunitarios ^43^, conductas de búsqueda de atención médica ^44^ o enfoque de género, como es el caso del enfoque de género en el Programa de Prevención y Atención a la Diabetes ^45^. En cambio, los adultos mayores, con mayor prevalencia y acumulación de factores de riesgo, muestran menor adopción de medidas preventivas ^46^ ^,^ ^47^.

Estas diferencias demográficas demandan estrategias adaptadas: políticas focalizadas en poblaciones de mayor riesgo y medidas que aseguren la continuidad y la intensidad de las intervenciones en el tiempo. De esta manera, vincular la estrategia con iniciativas de salud digital y fortalecimiento de la atención primaria, podría mejorar su sostenibilidad ^48^.

En conjunto, los resultados deben interpretarse como asociaciones temporales a nivel poblacional y no como evidencia de una relación causal directa entre la ENPCSOD y las variaciones observadas en la incidencia de diabetes mellitus tipo 2. Estas asociaciones deben entenderse en el marco de una política pública compleja, implementada de forma progresiva y coexistente con otras intervenciones y cambios estructurales.

Este estudio presenta limitaciones inherentes a su diseño ecológico y al uso de fuentes administrativas de notificación, las cuales pueden reflejar variaciones en el registro, el acceso a los servicios de salud y los procesos diagnósticos. El análisis ITS estima asociaciones temporales agregadas y asume un contrafactual lineal en ausencia de intervención; la coexistencia de políticas concurrentes o cambios sociodemográficos no modelados podría sesgar las estimaciones. Asimismo, la agregación anual de los datos impide identificar efectos estacionales o de corto plazo. Dada la larga latencia de la diabetes mellitus tipo 2, los cambios inmediatos observados no deben interpretarse como efectos biológicos directos de la política, sino como asociaciones influenciadas por factores operativos e institucionales. Futuras evaluaciones deberían incorporar comparadores internos, outcomes de control y modelos alternativos para fortalecer la inferencia.

En conclusión, la evaluación muestra que la ENPCSOD generó un efecto inmediato en la reducción de la incidencia de diabetes mellitus tipo 2 en adultos mexicanos, pero no logró sostenerlo a través del tiempo. Más allá de la interpretación biomédica, estos hallazgos subrayan la importancia de la gobernanza intersectorial, la sostenibilidad política y la integración social como condiciones necesarias para que las estrategias nacionales de prevención logren impactos poblacionales duraderos.

## Data Availability

Las bases de datos utilizadas en el estudio, incluyendo los códigos de extracción, los análisis y los resultados, están disponibles en el repositorio: https://epidemiologia.salud.gob.mx/anuario/html/index.html; https://www.datos.gob.mx/dataset/proyecciones-de-poblacion/resource/3c3092be-583e-4490-8c23-67ef9a64b198.

## Material Suplementar

Material Suplementar
