# Supplementary material for: Impact of the national strategy against obesity and diabetes on the incidence of diabetes in Mexico, 2005-2023
Source: Cad Saude Publica. 2026 Jul 27;42:e00180725. [Article in Spanish] doi: 10.1590/0102-311XES180725 (PMC13411465; doi:10.1590/0102-311XES180725)
Supplement: Material Suplementar [file 1678-4464-csp-42-ES180725-s.pdf]

Material suplementario

Cuadro S1 Publicación y entrada en vigor de las regulaciones para la prevención y el control del sobrepeso, la obesidad y la diabetes.

REGULACIÓN	PUBLICACIÓN EN DOF	ENTRADA EN VIGOR
Publicidad de alimentos y bebidas dirigida a niños.	15 de abril 2014	Se realizó en 3 momentos:<br>• Al día siguiente de la publicación.<br>• A los 90 días, es decir 15 de julio 2014 (botanas, bebidas saborizadas, chocolates, productos similares a chocolates y productos de confitería).<br>• El 1 de enero 2015 (productos no señalados).
Etiquetado frontal de alimentos y bebidas no alcohólicas.	15 de abril 2014.<br>25 de julio 2014 se modifica.	30 de junio de 2015.
Distintivo nutrimental.	15 de abril 2014.<br>25 de julio 2014 se modifica.	30 de junio de 2015.
Expendio y distribución de Alimentos y Bebidas en Escuelas.	23 de agosto de 2010.<br>16 de mayo 2014 se modifica.	3 etapas de implementación, una por ciclo escolar. En las escuelas del tipo básico: a partir del ciclo escolar 2014-2015. En escuelas del tipo medio superior y superior: el ciclo lectivo, el segundo semestre de 2014.
Impuestos a bebidas azucaradas y alimentos con alta densidad energética	11 de diciembre 2013.	1 enero 2014.

Tabla S1 Diagnósticos estadísticos del modelo_gls general.

Diagnóstico	Estadístico	Valor	p	Criterio	Cumple
Ljung-Box (lag 5)	Q	9,46	0,092	p≥0,05	Si
Ljung-Box (lag 10)	Q	14,85	0,138	p≥0,05	Si
Breusch-Pagan	χ^2	4,08	0,539	p≥0,05	Si
Normalidad (Q–Q)	-	-	-	alineación visual	Si
ACF	-	-	-	sin picos >	Si

Figura S1 Análisis de residuos del modelo_gls general.

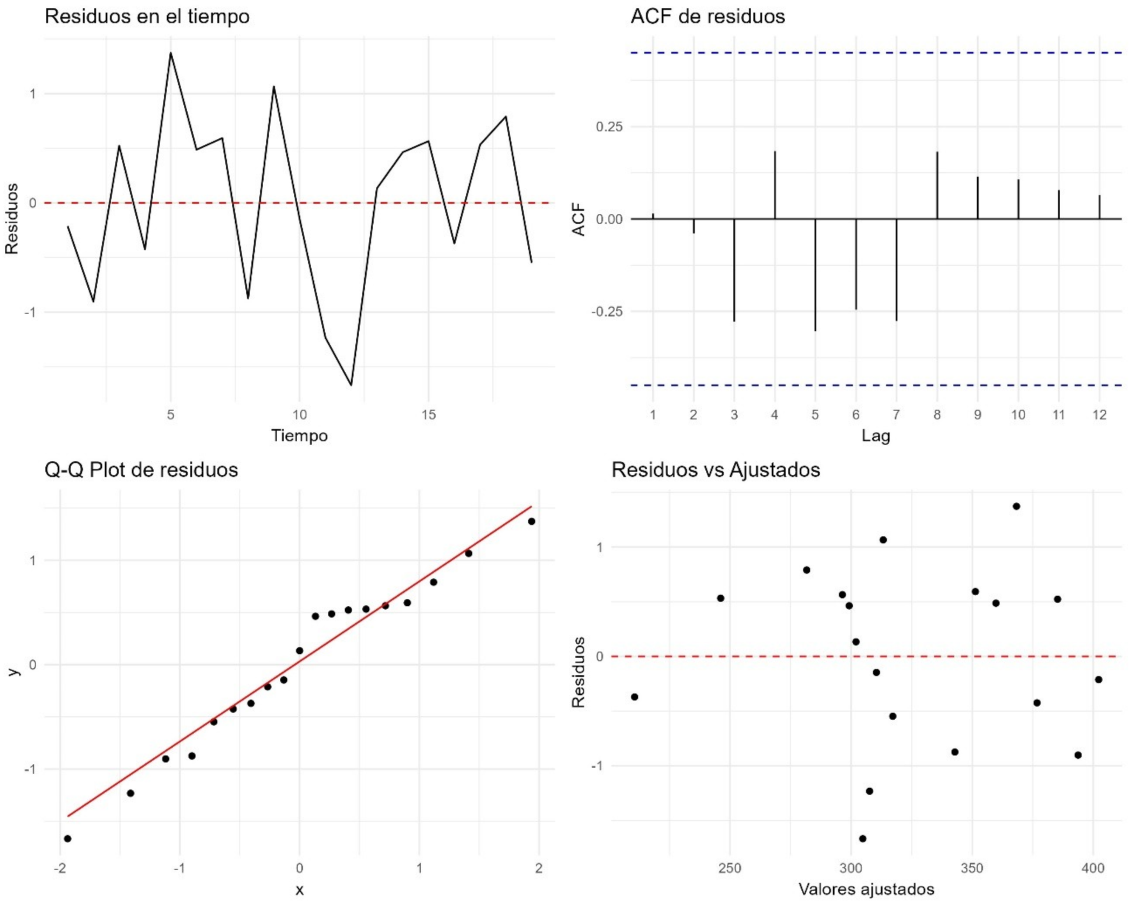

Tabla S2 Diagnósticos estadísticos del modelo_gls por grupos de edad.

Diagnóstico	Estadístico	Valor	p	Criterio	Cumple
Ljung-Box (lag 5)	Q	10,37	0,065	$p \geq 0,05$	Si
Ljung-Box (lag 10)	Q	13,36	0,204	$p \geq 0,05$	Si
Breusch-Pagan	χ^2	1,16	0,949	$p \geq 0,05$	Si
Normalidad (Q–Q)	-	-	-	alineación visual	Si
ACF	-	-	-	sin picos >	Si

Figura S2 Análisis de residuos del modelo_gls por grupos de edad.

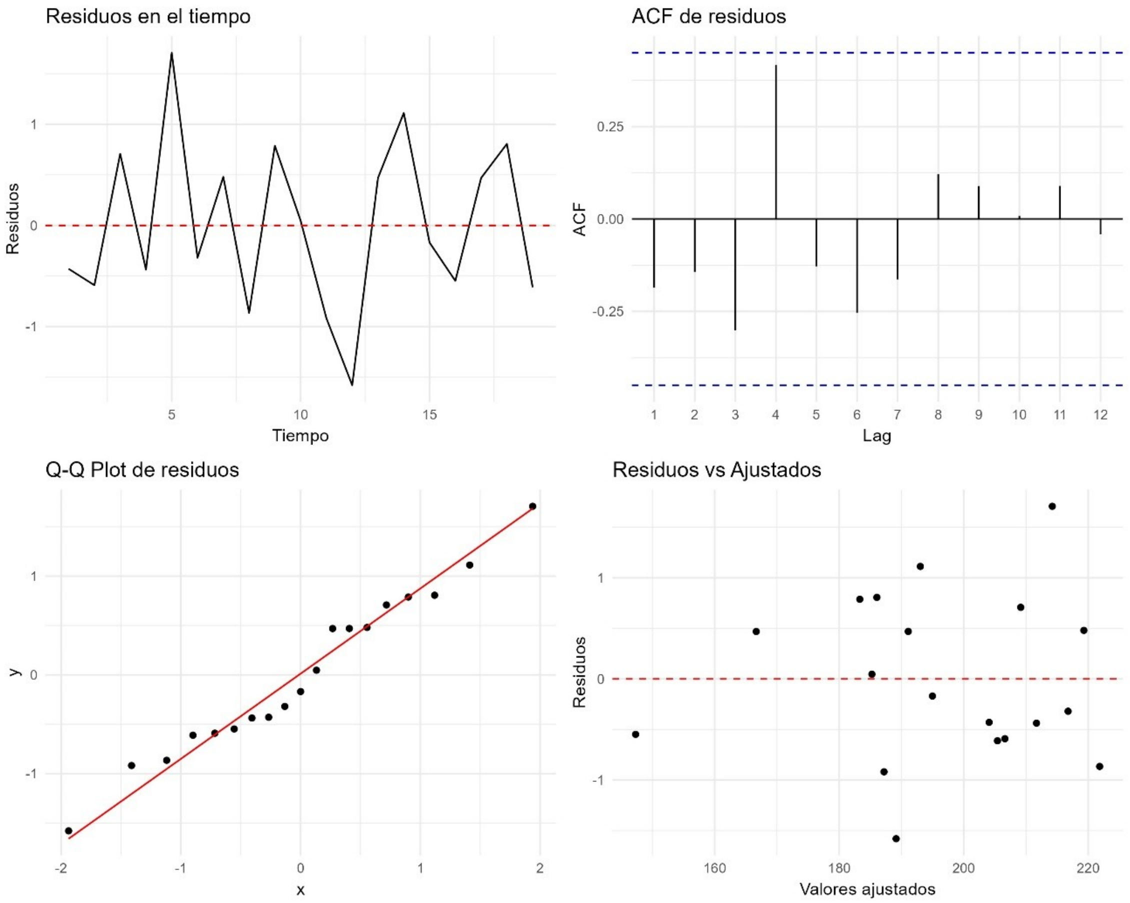

Tabla S3 Diagnósticos estadísticos del modelo_gls por sexo.

Diagnóstico	Estadístico	Valor	p	Criterio	Cumple
Ljung-Box (lag 5)	Q	8,86	0,112	$p \geq 0,05$	Si
Ljung-Box (lag 10)	Q	13,35	0,205	$p \geq 0,05$	Si
Breusch-Pagan	χ^2	2,92	0,712	$p \geq 0,05$	Si
Normalidad (Q–Q)	-	-	-	alineación visual	Si
ACF	-	-	-	sin picos >	Si

Figura S3 Análisis de residuos del modelo_gls por sexo.

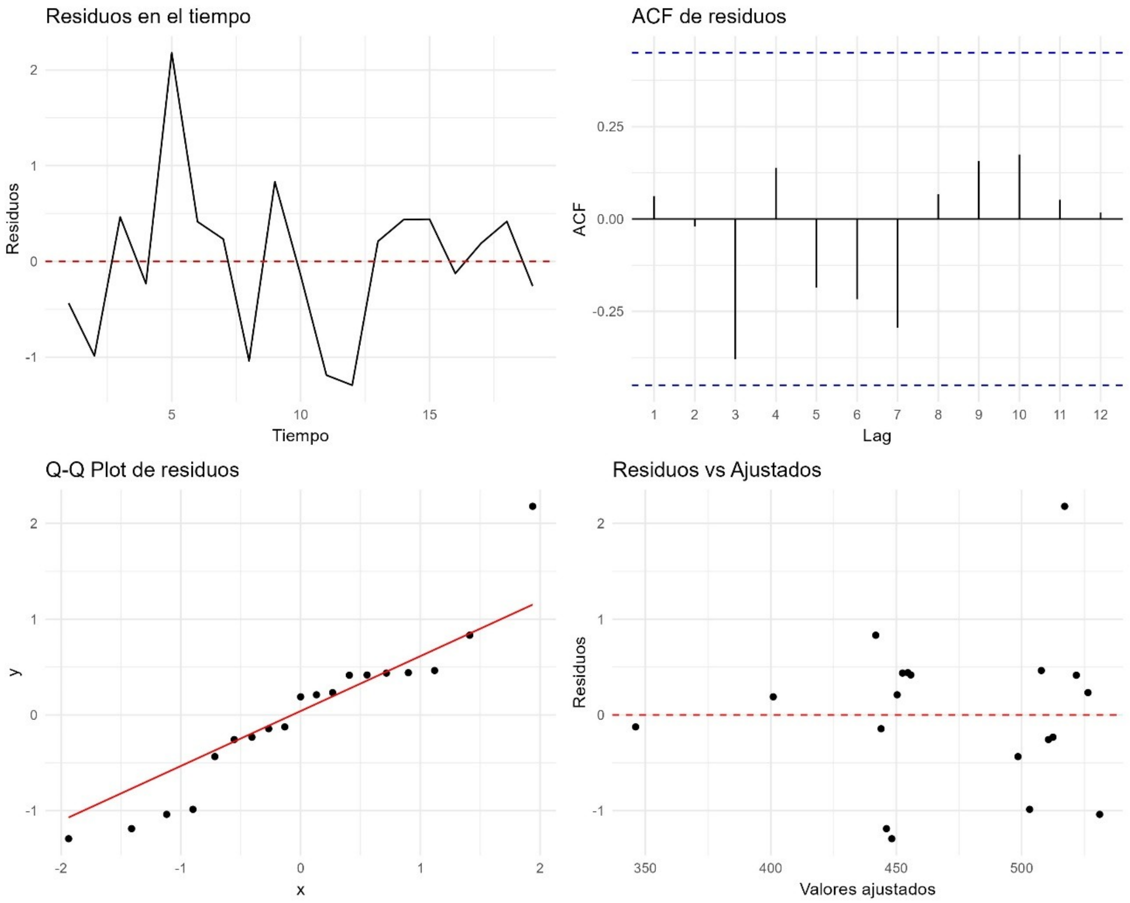

Tabla S4 Impacto absoluto y relativo en la incidencia de diabetes mellitus tipo 2 general, por edad y sexo.

	Impacto absoluto ENPCSOD (2013)	Impacto relativo ENPCSOD (2013)	Impacto absoluto COVID-19 (2020)	Impacto relativo COVID-19 (2020)
General				
	-59,44	-16,62%	-73,63	-25,44%
Grupos de edad				
20-44	-44,03	-19,37%	-52,72	-26,36%
45-59	-156,99	-13,99%	-233,80	-25,52%
60 y más	-194,36	-14,62%	-261,78	-25,36%
Sexo				
Masculino	-102,53	-18,83%	-120,19	-25,78%
Femenino	-98,03	-14,70%	-148,31	-25,87%

Tabla S5 Análisis de sensibilidad para el modelo_gls general.

Coeficiente	Estimación	Error estándar	t	p	IC 95 % inferior	IC 95 % superior
Intercepto	412,12	12,32	33,45	0,000	387,97	436,27
Tiempo	-7,08	2,41	-2,93	0,014	-11,81	-2,35
Intervención ENPCSOD	-55,37	15,98	-3,46	0,005	-86,69	-24,04
Pendiente post-ENPCSOD	5,30	3,91	1,36	0,202	-2,35	12,96
Intervención COVID-19	-40,78	64,98	-0,63	0,543	-168,15	86,59
Pendiente post-COVID-19	19,37	18,28	1,06	0,312	-16,46	55,19

Tabla S6 Análisis de sensibilidad para el modelo_gls por grupos de edad.

	Coeficiente	Estimación	Error estándar	t	p	IC 95 % inferior	IC 95 % superior
**Grupos de edad**							
20-44	Intercepto	201,34	6,70	30,05	0,000	188,21	214,47
	Tiempo	2,61	1,32	1,97	0,074	0,02	5,21
	Intervención ENPCSOD	-41,41	9,03	-4,58	0,001	-59,12	-23,70
	Pendiente post-ENPCSOD	-0,59	2,10	-0,28	0,786	-4,71	3,54
	Intervención COVID-19	-24,81	41,01	-0,61	0,557	-105,20	55,57
	Pendiente post-COVID-19	5,50	11,58	0,47	0,644	-17,20	28,20
45-59	Intercepto	1310,50	38,90	33,69	0,000	1234,25	1386,75
	Tiempo	-23,16	7,63	-3,04	0,011	-38,11	-8,21
	Intervención ENPCSOD	-152,20	50,67	-3,00	0,012	-251,51	-52,90
	Pendiente post-ENPCSOD	15,03	12,32	1,22	0,248	-9,12	39,18
	Intervención COVID-20	-173,04	207,84	-0,83	0,423	-580,41	234,33
	Pendiente post-COVID-20	71,68	58,48	1,23	0,246	-42,95	186,31
60+	Intercepto	1820,51	61,37	29,66	0,000	1700,23	1940,80
	Tiempo	-57,74	11,92	-4,84	0,001	-81,10	-34,37
	Intervención ENPCSOD	-204,24	76,29	-2,68	0,022	-353,76	-54,71
	Pendiente post-ENPCSOD	40,76	19,61	2,08	0,062	2,33	79,19
	Intervención COVID-21	-96,57	290,18	-0,33	0,746	-665,31	472,18
	Pendiente post-COVID-21	87,58	81,27	1,08	0,304	-71,71	246,87

Tabla S7 Análisis de sensibilidad para el modelo_gls por sexo.

Sexo	Coeficiente	Estimación	Error estándar	t	p	IC 95 % inferior	IC 95 % superior
Femenino	Intercepto	764,43	21,36	35,79	0,000	722,56	806,29
	Tiempo	-12,60	4,18	-3,02	0,012	-20,78	-4,41
	Intervención ENPCSOD	-94,73	27,45	-3,45	0,005	-148,53	-40,93
	Pendiente post-ENPCSOD	12,68	6,78	1,87	0,088	-0,61	25,98
	Intervención COVID-21	-56,45	109,48	-0,52	0,616	-271,04	158,13
	Pendiente post-COVID-21	36,99	30,76	1,20	0,254	-23,30	97,29
Masculino	Intercepto	493,49	24,31	20,30	0,000	445,84	541,14
	Tiempo	4,76	4,76	1,00	0,338	-4,56	14,09
	Intervención ENPCSOD	-92,13	31,45	-2,93	0,014	-153,77	-30,49
	Pendiente post-ENPCSOD	-2,69	7,71	-0,35	0,734	-17,80	12,43
	Intervención COVID-21	-98,80	127,09	-0,78	0,453	-347,90	150,29
	Pendiente post-COVID-21	35,09	35,74	0,98	0,347	-34,95	105,13
